# Pseudoaneurysm of the Mesenteric Artery Status Post Drain Placement for Complicated Diverticulitis

**DOI:** 10.7759/cureus.12394

**Published:** 2020-12-31

**Authors:** Brianna L Spencer, Michael Deutsch

**Affiliations:** 1 Surgery, Penn State Health Milton S. Hershey Medical Center, Hershey, USA; 2 Colorectal Surgery, Penn State Health Milton S. Hershey Medical Center, Hershey, USA

**Keywords:** complicated diverticulitis, diverticulitis, colon abscess, ct guided drainage, pseudoaneurysm

## Abstract

Perforated diverticulitis presents a challenging clinical scenario for the surgeon. Development of an abscess in those without an acute abdomen may be amendable to non-operative drainage. Furthermore, early intervention can dramatically alter the hospital course let alone the overall outcome. While relatively safe as a procedure, image-guided drainage does carry risk that needs to be calculated relative to benefit gained. One rare albeit possibly serious risk is pseudoaneurysm formation.

## Introduction

CT-guided drainage for abscess formation after acute perforated diverticulitis is a treatment option for those patients without an acute abdomen who present with a fluid collection or abscess that can be reached safely through imaging. Early drainage can improve a patient’s clinical course by converting an emergent surgical situation into an elective one. Percutaneous drainage also reduces septic time, stoma creation, and decreasing hospital stay [1,2]. Technical success is directly apparent with purulent contents and success rates upwards of 90% [3]. However, any intervention carries potential harm that can overshadow its benefit. Typical complications involve pain, sedation sequella, worsening of sepsis, and non-target catheter placement, among others. Pseudoaneurysm is also a well-established risk of percutaneous drainage procedures; however, this rate is very low [4]. Furthermore, when reported in the literature, it tends to occur with drainage of high-risk vascular structures such as the liver or spleen. We present a unique case of CT-guided drainage for a diverticular abscess and subsequent development of a pseudoaneurysm one day following drainage.

## Case presentation

**Figure 1:**
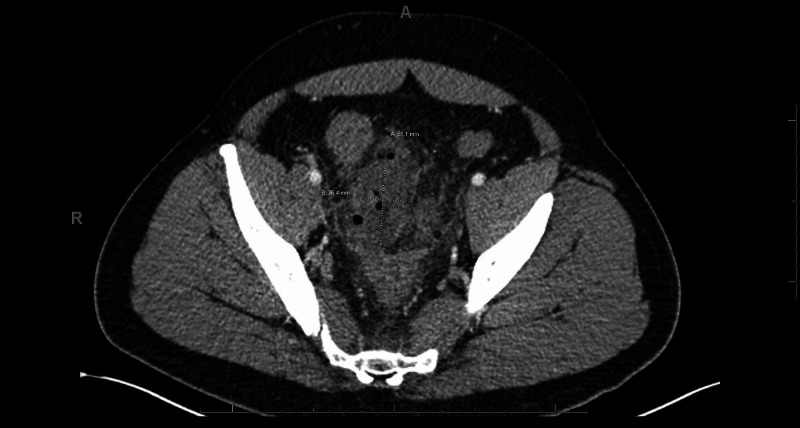
Organized collection

We present the case of a 35-year-old male with a past medical history significant for asthma who presented to our hospital ED with abdominal pain. He was admitted one week prior for acute diverticulitis with microperforation, received IV antibiotics, and was discharged with plans to transition to an oral antibiotic regiment. Two days following discharge, he experienced increased abdominal pain that continued to worsen radiating to his lower back. While in the ED on his second admission, he was febrile to 39 degrees Celsius, with WBC of 18,000/mm3 and lactate level of 1 mmol/L; erythrocyte sedimentation rate (ESR) and C-reactive protein (CRP) were not obtained at this time. He had a repeat CT scan that demonstrated an organized collection measuring 3.6 x 6.5 cm (Figure 1).

**Figure 2:**
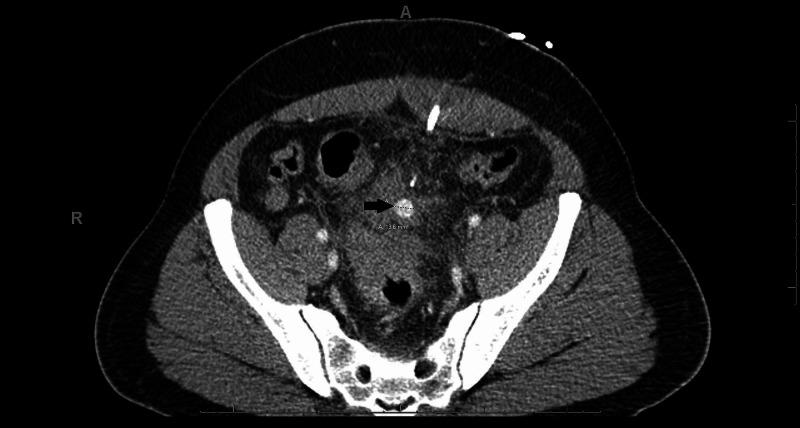
Pseudoaneurysm

He underwent CT-guided drainage with cultures and had symptomatic improvement. Three days following drainage, his output suddenly changed from purulent to bloody, accompanied by multiple bloody bowel movements with increased abdominal pain. An urgent CT with contrast was performed, which was remarkable for a mesenteric artery pseudoaneurysm near the site of the drain placement (Figure 2).

**Figure 3:**
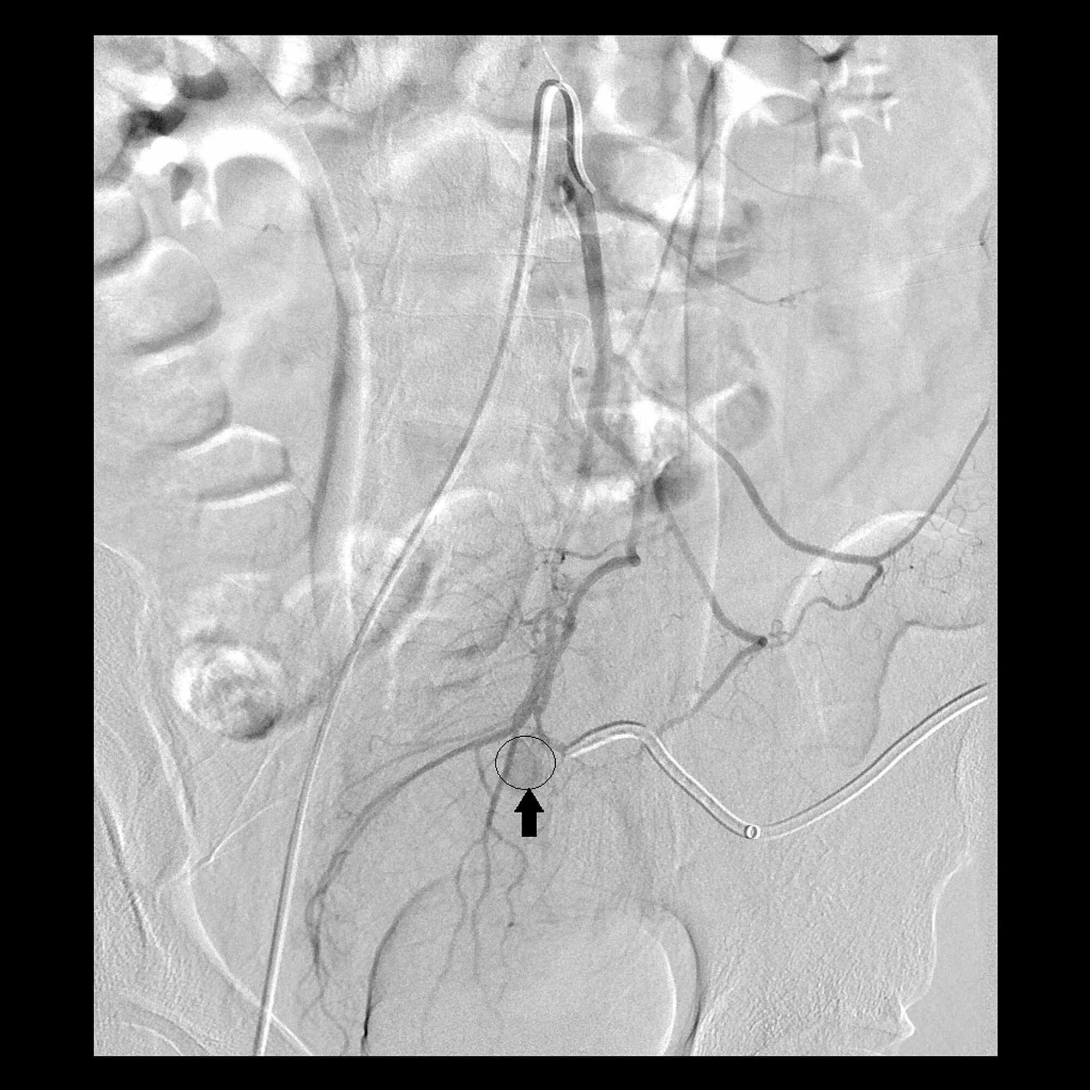
Interventional radiology image pre-coil embolization

Once again, interventional radiology service was consulted and coil and foam embolization was performed (Figure 3). He did well afterwards and was able to undergo a laparoscopic low anterior resection upon optimization three days later. He has since recovered well without additional sequella.

## Discussion

CT-guided percutaneous drainage in patients with acute complicated diverticulitis with abscess formation is a well-documented form of sepsis/infection management. While it is well described and performed daily, it is not without risk. It is important to consider the size of the abscess in the risk/ benefit of drainage [5]. Common risks include but are not limited to bleeding, sepsis, inappropriate placement of catheter, post-procedural pain, and aneurysm formation. Infection/sepsis can occur due to placement of the catheter itself seeding infection. Sepsis can also worsen if the catheter is placed in the wrong location such as the bowel. Infectious spread of contents to the bloodstream occurs in 5% of cases [3]. The collections that are drained may also be in close proximity to vascular structures, which can lead to the complication of bleeding or pseudoaneurysm formation after drain placement.

It is important when selecting patients for percutaneous drainage to recognize the risk factors associated with their development. In the case of this procedure, pseudoaneurysm formation is a rare complication. Given that this is a rare complication from percutaneous drainage, there is a paucity of information available in the literature on management and treatment. Although pseudoaneurysm risk is low, it is important to consider and to be aware of the potential presenting symptoms following drain placement. Endovascular repair in the literature available is the preferred method of management, as open repair comes with increased morbidity and mortality [6,7]. In a case report of super mesenteric artery pseudoaneurysm following Whipple, a patient underwent endovascular repair with stent placement with good outcomes [8]. Options for endovascular repair include stent placement, coil embolization, or foam embolization. Endovascular repair has been shown to have decreased morbidity and decreased time to recovery.

Our patient recovered well following drain removal and coil embolization, but pseudoaneurysm rupture can lead to serious complications. It is imperative to recognize aneurysms early to avoid the complication of rupture and bleeding.

## Conclusions

While image-guided drainage procedures for abscess following complicated diverticular disease are generally well tolerated, short and possible long-term complications may ensue. To battle these, the surgeon should perform a thorough review of the pre-intervention imaging and associated comorbidities of the patient, taking particular care to assess size, location, and means of the fluid collection. Complimenting these measures, a detailed discussion of complications, albeit rare ones such as pseudoaneurysm, should occur in the consent process. Then, when complications do occur, rapid identification and action on the cause can help mitigate the potential long-term consequences.
